# SARS-CoV-2 nsp1 mediates broad inhibition of translation in mammals

**DOI:** 10.1016/j.celrep.2025.115696

**Published:** 2025-05-12

**Authors:** Risako Gen, Amin Addetia, Daniel Asarnow, Young-Jun Park, Joel Quispe, Matthew C. Chan, Jack T. Brown, Jimin Lee, Melody G. Campbell, Christopher P. Lapointe, David Veesler

**Affiliations:** 1 Department of Biochemistry, University of Washington; Seattle, WA 98195, USA.; 2 Division of Basic Sciences, Fred Hutchinson Cancer Center, Seattle, WA, USA.; 3 Howard Hughes Medical Institute, University of Washington; Seattle, WA 98195, USA.; 4 Lead contact

## Abstract

SARS-CoV-2 nonstructural protein 1 (nsp1) promotes innate immune evasion by inhibiting host translation in human cells. However, the role of nsp1 in other host species remains elusive, especially in bats - natural reservoirs of sarbecoviruses with a markedly different innate immune system than humans. We reveal that nsp1 potently inhibits translation in *Rhinolophus lepidus* bat cells, belonging to the same genus as known sarbecovirus reservoirs hosts. We determined a cryo-electron microscopy structure of nsp1 bound to the *R. lepidus* 40S ribosomal subunit, showing that it blocks the mRNA entry channel by targeting a highly conserved site among mammals. Accordingly, we found that nsp1 blocked protein translation in mammalian cells from several species, underscoring its broadly inhibitory activity and conserved role in numerous SARS-CoV-2 hosts. Our findings illuminate the arms race between coronaviruses and mammalian host immunity, providing a foundation for understanding the determinants of viral maintenance in bat hosts and spillover.

## Graphical Abstract

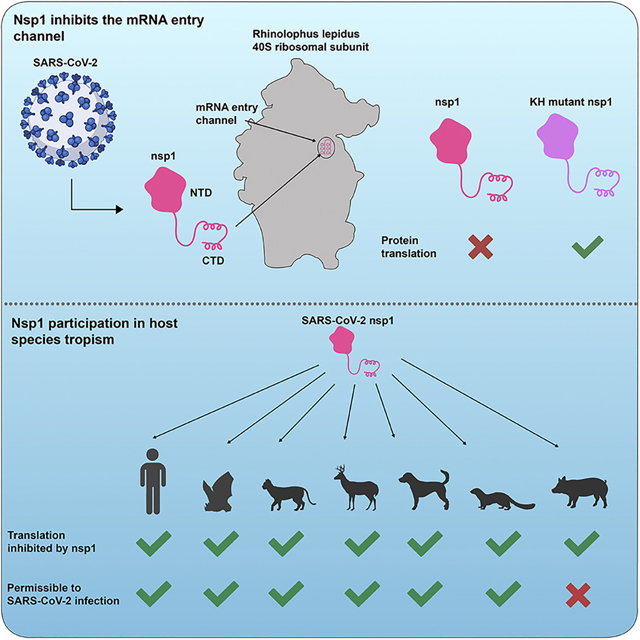

## Introduction

Outbreaks of disease have plagued humans since the beginning of recorded history. Around 75% of human infectious diseases are thought to originate from spillover of non-human animal pathogens, also known as zoonosis^1^. Climate change, urbanization, and globalization all contribute to the increasing frequency with which new pathogens are introduced into the human population as a result of interactions between animals and humans^2^. Although many wild animals are natural reservoirs of pathogens, bats (Chiropteran order) are unusual in their ability to harbor more diverse viruses per animal species than other animals^3,4^. Recent studies have suggested that unique characteristics of the bat innate immune system allow them to tolerate viruses that are highly pathogenic to humans and other animals, such as coronaviruses, paramyxoviruses, and filoviruses^5–15^. While prior research has improved our understanding of the antiviral response in select bat species, there is a scarcity of commercially-available tools and reagents specific to bats which are not model organisms^16–21^. Given the increasing number of spillover events of bat viruses to humans, there is a need to study the mechanisms of viral tolerance in bats to better predict and control future viral spillovers.

Within the past two decades, three coronaviruses with ancestral origins in bats have caused major outbreaks: severe acute respiratory syndrome coronavirus (SARS-CoV) in 2002, Middle East respiratory syndrome coronavirus (MERS-CoV) in 2012, and SARS-CoV-2 in 2019^22–26,^. SARS-CoV-2 is a positive-sense single-stranded RNA virus that is part of the Betacoronavirus genus and Coronaviridae family^27^. The approximately 30 kb genome consists of 14 open reading frames (ORFs), of which ORF1a and ORF1b at the 5’ end are directly translated into two polyproteins by cellular ribosomes and processed to produce sixteen nonstructural proteins designated nsp1-nsp16^28^. These nsps work in unison and interfere with host cellular function in different ways to promote viral propagation.

Nsp1 is a major virulence factor of SARS-CoV-2 that antagonizes the host innate immune system through inhibition of host protein translation, blockage of mRNA nuclear export, promotion of host mRNA cleavage, and depletion of key host signaling factors^29–31^. Translation inhibition occurs through binding of the nsp1 C-terminal domain (CTD) to the mRNA entry channel within the human small ribosomal subunit (40S), sterically blocking host mRNA from entering the ribosome^29,32,33^. Whereas this leads to suppression of host mRNA translation, including the interferon response, viral mRNAs evade this nsp1-mediated translation inhibition through an unknown mechanism thought to include key interactions between the nsp1 N-terminal domain (NTD) and the SARS-CoV-2 mRNA 5’ untranslated region (UTR)^34,35^. Nsp1 mediates similar biological functions across alpha- and betacoronaviruses^36–38^, and three beta-CoV nsp1s were shown to inhibit translation through binding to the human 40S ribosomal subunit^39,40^, highlighting its importance as a conserved virulence factor.

Beyond its direct impact on the global human population, SARS-CoV-2 infections have been reported in many other animals such as bats, dogs, cats, minks, and deers, showcasing its broad host tropism^41–44^. While viral entry into host cells relies on receptor engagement, the success of viral replication is dependent on the virus’s ability to hijack the infected host cell, as illustrated by the fact that although pig ACE2 supports spike-mediated cell entry, pigs are refractory to SARS-CoV-2 infection^45–48^. These results underscore the importance of identifying key viral determinants of successful SARS-CoV-2 replication in host animals. Given the poor understanding of the bat innate immune system and the marked differences from the human counterpart that emerged from prior studies, the virus-host dynamics leading to reservoir maintenance and spillovers remain elusive.

We set out to understand the impact of SARS-CoV-2 nsp1 on the innate immune response of bats and other mammals to get deeper insight into nsp1 function across diverse host species. Here, we unveil the architecture of the *Rhinolophus lepidus* 40S ribosomal subunit, demonstrate the broad translation inhibition activity mediated by nsp1 in bats and other mammalian species, and reveal a conserved binding mechanism of nsp1 to the *R. lepidus* ribosome. Our findings provide a foundation for understanding the determinants of nsp1-induced host innate immune antagonism and spillovers in a wide range of mammals.

## Results

### SARS-CoV-2 nsp1 inhibits translation in *Rhinolophus lepidus* bat cells

**Fig 1.**
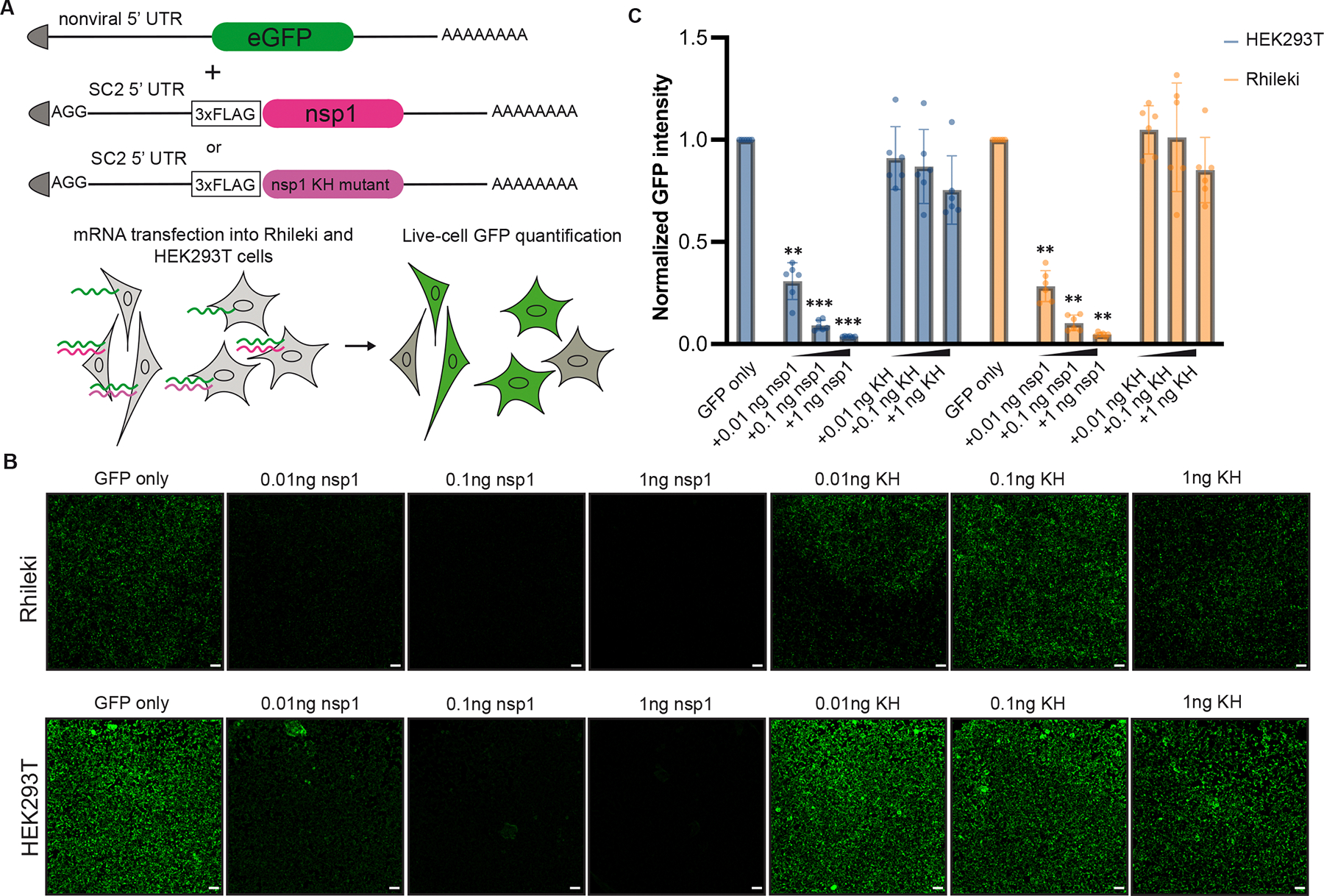
SARS-CoV-2 nsp1 suppresses translation in human (HEK293T) and Rhileki (bat) cells. (A) mRNA construct design and schematic of the fluorescence-based translation assay. 200 ng of eGFP-encoding mRNA was transfected with 0.01–1 ng of a wildtype or K164A/H165A double mutant nsp1 mRNA (KH) to enable visualization and quantification of translation inhibition. mRNAs are all 5’ capped (shown by the triangular grey shapes) and have poly(A) tails at the 3’ end. SC2: SARS-CoV-2. 5’ UTR: 5’ untranslated region. 3xFLAG: triple flag tag. KH: nsp1 K164A/H165A double mutant. (B) Live-cell fluorescence imaging 16h post transfection of GFP expression in the presence (or absence) of varying amounts of wildtype or KH nsp1 using Rhileki and HEK293T cells. Scale bar: 100 μm. (C) Quantification of the nsp1-mediated dose-dependent inhibition of translation 16h post transfection based on normalized GFP fluorescence intensity across the entire field of view. Bars represent the mean of six biological replicates shown as individual data points with error bars showing standard deviation. Each biological replicate is a mean of six technical replicates. Transfections with wildtype and mutant nsp1 mRNAs were compared to the GFP-only control using one-way ANOVA and follow-up Dunnet’s T3 multiple comparisons tests. P-values reported are adjusted for multiplicity. **: p<0.01, ***: p<0.005.

To evaluate if SARS-CoV-2 nsp1 is functional in bat cells, we investigated nsp1-mediated inhibition of translation in a *Rhinolophus lepidus* kidney (Rhileki) cell line, which we selected as a model of sarbecovirus target cells given that Rhinolophus horseshoe bats act as natural reservoirs of SARS-CoV-2-related coronaviruses^49,50^. Moreover, the Rhileki cell line we use sustains SARS-CoV-2 replication (albeit weakly) and cytopathic effects, making it an ideal and relevant choice^51^. To quantify the impact of nsp1 activity on protein synthesis, we developed a fluorescence-based translation assay involving nsp1 and GFP reporter mRNAs (Fig 1A). The mRNA constructs are capped at the 5’ end and harbor a 80-nt poly-A tail at the 3’ end to mimic mature mRNAs, and contain N1-methyl pseudouridines (instead of uridines) as it was shown to dampen innate immune activation in humans cells^52^. We chose to include a synthetic non-viral 5’ UTR for the GFP-encoding mRNA whereas the nsp1-encoding mRNA possesses the SARS-CoV-2 5’ UTR sequence to overcome nsp1-mediated translation inhibition^34,39^. Transfection of the GFP mRNA in both Rhileki and HEK293T cells led to robust expression observed by live-cell fluorescence imaging (Fig 1B). Co-transfection of the GFP and wildtype nsp1 mRNAs reduced fluorescence in an nsp1 mRNA dose-dependent manner with 75% reduction of GFP expression achieved with 0.01 ng of nsp1 mRNA and no detectable fluorescence remained with transfection of 1 ng of nsp1 mRNA in both cell lines (Fig 1C). Furthermore, the previously described nsp1 K164A/H165A double mutant^53^, which is unable to inhibit human ribosome-mediated translation, had no effect on GFP translation in Rhileki or HEK293T cells (Fig 1C and S1B). As *Rhinolophus lepidus* is part of the Yinpterochiroptera suborder, we evaluated the impact of nsp1 in *Tadarida brasiliensis* cells, a species that is part of the Yangochiroptera suborder, thought to have evolutionarily diverged around 63 million years ago^54,55^. Transfections conducted in a *T. brasiliensis* lung (Tb 1 Lu) cell line demonstrated that nsp1 dampened GFP expression in this divergent bat species (Fig S2). These results indicate that nsp1 inhibits protein translation in human, *R. lepidus*, and *T. brasiliensis* cells.

### Architecture of the *Rhinolophus lepidus* bat ribosome

**Figure 2.**
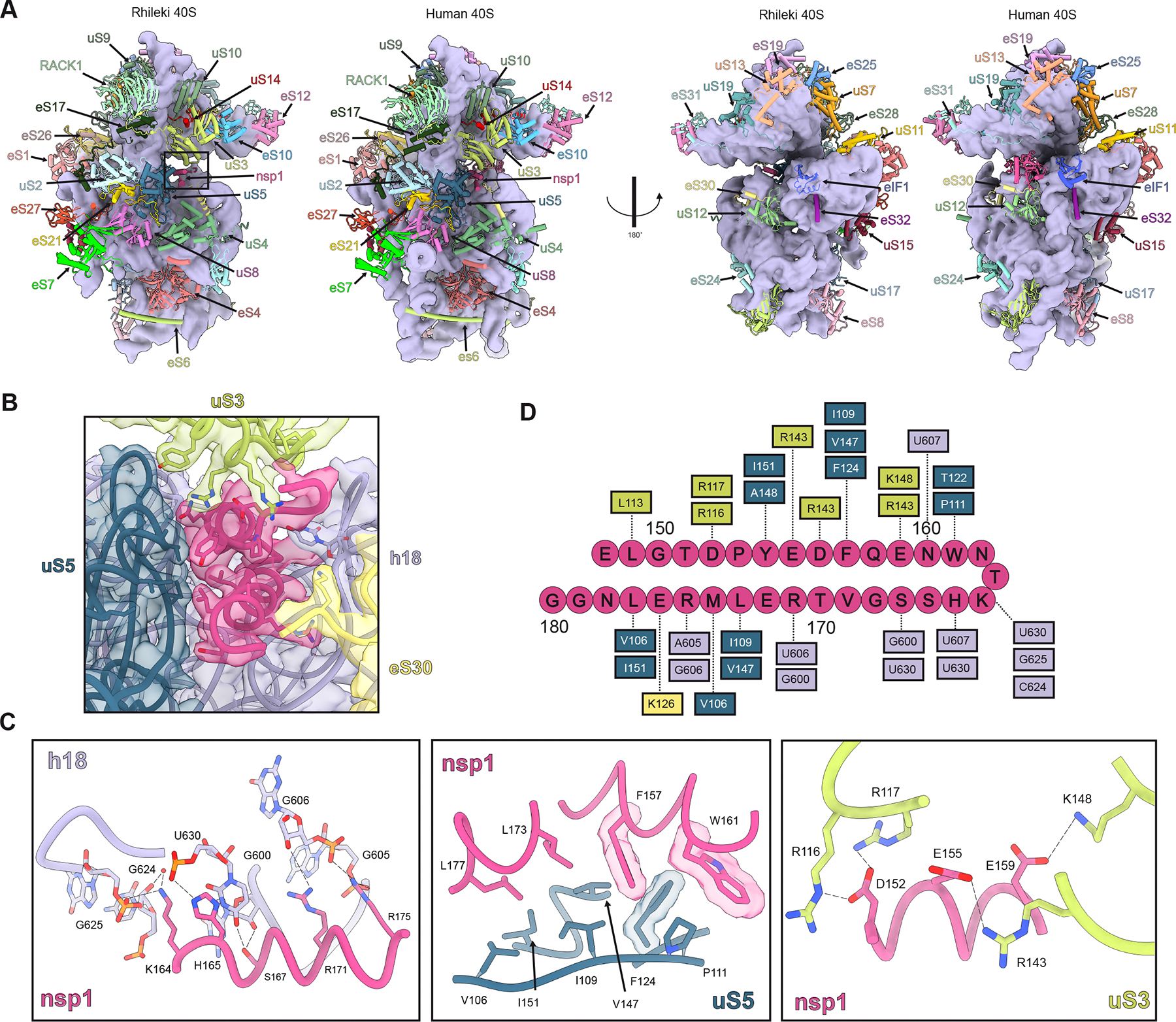
Architecture of the Rhileki bat 40S ribosomal subunit bound to SARS-CoV-2 nsp1. (A) Comparison of the Rhileki and human 40S ribosomal subunit organization (PDB: 8PPK). Each ribosomal protein is colored distinctly and labeled whereas the 18S rRNA is rendered as a purple surface. The box indicates the nsp1 binding interface shown in panel B. (B) Closeup view of nsp1 (pink) within the mRNA entry channel of the Rhileki 40S ribosomal subunit and its interacting partners uS5 (blue), uS3 (light green), eS30 (yellow), and helix 18 (h18) of the 18S rRNA (purple). The model is fitted within the consensus cryo-EM map rendered as an isosurface. (C) Key interactions of nsp1 CTD with the 18S rRNA, uS5, and uS3. Polar interactions are shown as black dotted lines. Select residues are rendered with a semi-transparent surface. (D) Interaction map of the nsp1 CTD with the 40S ribosomal subunit with key ribosomal interactors shown through dotted lines.

Although prior studies have provided a wealth of information on several prokaryotic and eukaryotic ribosomes^56–63^, the architecture and composition of the Chiropteran ribosome remain elusive. To address this knowledge gap and understand the mechanism of action of nsp1 in bats, we sought to purify ribosomes from the Rhileki cell line. Fractionation of Rhileki cell lysates through a 30% sucrose cushion (to pellet the ribosomes) followed by purification using a 11–25% sucrose density gradient enabled the isolation of pure 40S ribosomal subunit (Fig S3A–B). The purified 40S ribosomal subunit was subsequently incubated with recombinantly produced SARS-CoV-2 nsp1 and human eIF1 (Fig S3C–D) at a 1:5:5 molar ratio before sample vitrification for structural analysis. We used single particle cryo-EM to determine a structure of the SARS-CoV-2 nsp1-bound Rhileki 40S ribosomal subunit at an overall resolution of 2.1 Å (Table S1). Subsequent local refinements yielded maps at 2.1 Å resolution with significant improvements in the dynamic regions of the 40S subunit, such as the 40S head (Fig S4). Recombinant human eIF1 was added to promote stabilization of the nsp1 N-terminal domain (NTD), as previously described^39^. Although the eIF1 density was partially resolved in the cryo-EM map, the nsp1 NTD density was too weak to be modeled. Given that there is no genomic information for Rhileki, we sequenced all ribosomal proteins via transcriptomics and used Sanger sequencing of the 18S rRNA and ribosomal protein S10 to enable model building of the entire 40S subunit (Table S1). Comparing the Rhileki 40S and human 40S ribosomal subunit structures reveals a remarkable architectural and compositional similarity (Fig 2A), concurring with the conservation of their ribosomal protein sequences (97–100% amino acid sequence identity) and of their 18S rRNA (99.52% nucleotide sequence identity). We note that the 60S ribosomal subunit protein sequences are less conserved than that of the 40S when comparing Rhileki to human ribosomes, with the lowest (85.1%) sequence identity for eL29 (Table S1). Our data show that the Rhileki 40S ribosomal subunit is strikingly similar to the human 40S subunit despite extensive evolutionary divergence between the Chiropteran and Primate orders, likely reflecting the strong selective pressure in maintaining the functionality of the machinery mediating protein translation.

### Molecular basis of SARS-CoV-2 nsp1-mediated Rhileki translation inhibition

Our cryo-EM structure resolves the nsp1 CTD embedded in the mRNA entry channel of the ribosome, confirming successful complex formation and enabling visualization of nsp1 recognition of the Rhileki 40S ribosomal subunit (Fig 2B). As seen in cryo-EM structures of nsp1 bound to the human ribosome, the CTD forms two alpha helices (consisting of residues 153–160 and 166–178) and interacts extensively with uS5, uS3, eS30, and helix h18 of the 18S rRNA (Fig 2C–D). Extensive van der Waals interactions are formed between nsp1 and uS5 involving nsp1 residues F157, W161, L173, and L177, and uS5 residues V106, P111, F124, V157, I151, and I190, along with aromatic stacking of nsp1 F157 and uS5 F124. Nsp1 helix 1 residues interact with uS3 through predominantly polar contacts with D152, E155, and E159 forming salt bridges with uS3 residues R116, R143, and K148, respectively. The 18S rRNA h18 forms multiple hydrogen bonds and electrostatic interactions with nsp1 helix 2. The phosphate groups of G606, G625, and U630 respectively interact with the positively charged side chains of R175, K164, and H165, whereas S167 is hydrogen-bonded to U630 and the G600 ribose. The conserved K164/H165 motif forms multiple contacts with U607, C624, G625, and U630, highlighting its importance in promoting CTD interactions within the Rhileki ribosome, thereby rationalizing the loss of function of the nsp1 K164A/H165A double mutant (Fig 1B–C). Overall, nsp1 buries 1655 Å^2^ of its surface at the interface with the 40S, showcasing very extensive interactions despite its relatively small size, concurring with its high binding affinity^40,64^. Collectively, our data show that the nsp1 CTD interacts with the Rhileki ribosome using a conserved binding mode to that observed for the human ribosome^29,32,33^, leading to translation inhibition via blockade of the mRNA entry channel.

### Nsp1 inhibits translation in a broad range of SARS-CoV-2 hosts

**Figure 3.**
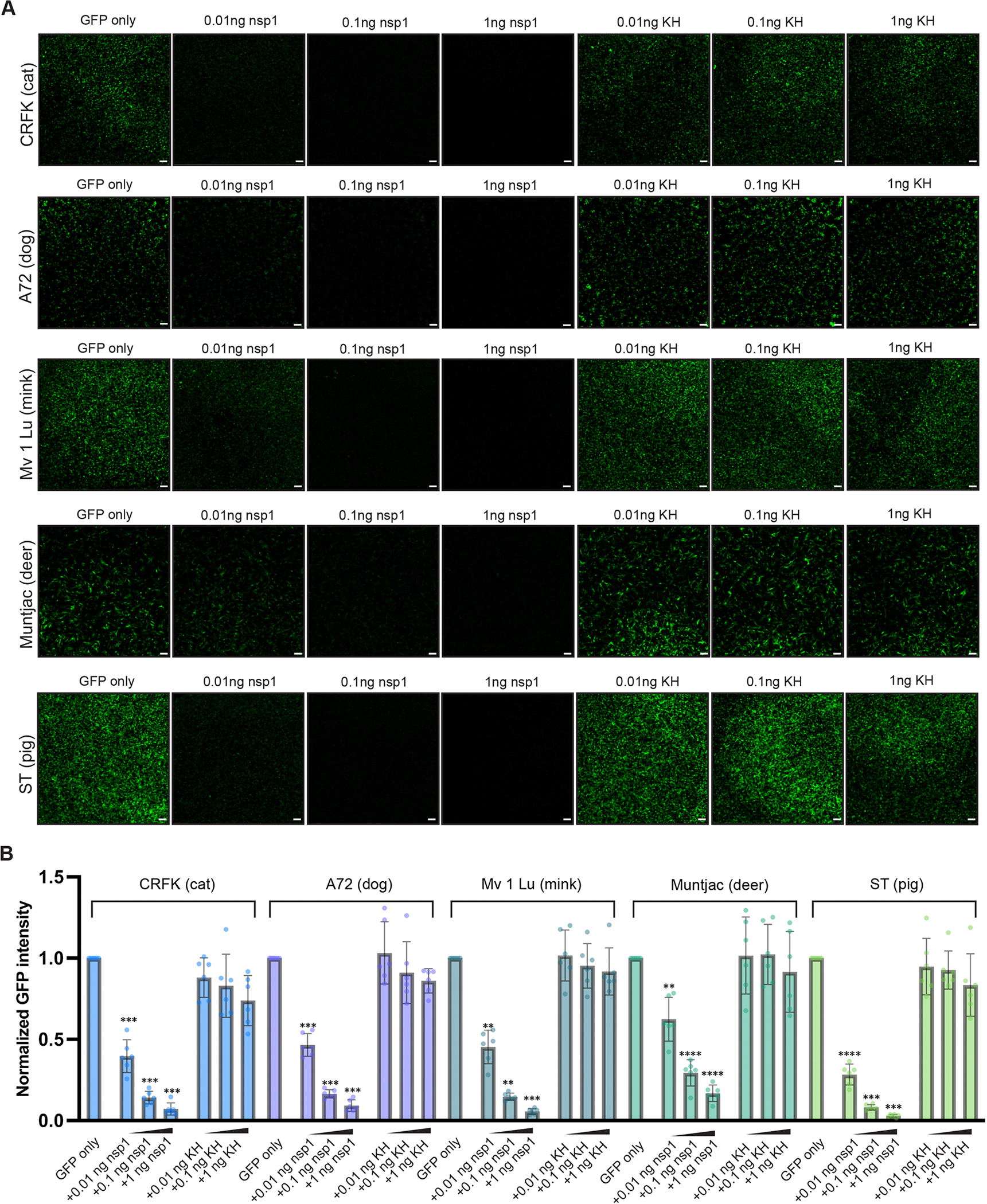
SARS-CoV-2 nsp1 inhibits translation in a wide range of mammalian species. (A) Live-cell fluorescence imaging of GFP expression in the presence (or absence) of varying amounts of wildtype or K164A/H165A mutant (KH) nsp1 mRNA in Crandell-Rees feline kidney epithelial cells (CRFK), canine fibroblasts cells (A72), American mink epithelial cells (Mv 1 Lu), Southern red muntjac deer fibroblast cells (Muntjac), and pig testis fibroblast cells (ST). Scale bar: 100 μm. (B) Quantification of the nsp1-mediated dose-dependent inhibition of translation based on normalized GFP fluorescence intensity across the entire field of view. Bars represent the mean of six biological replicates shown as individual points. Each biological replicate is a mean of six technical replicates. Transfection with wildtype and mutant nsp1 mRNAs were compared to the GFP-only control using one-way ANOVA and follow-up Dunnet’s T3 multiple comparisons tests. P-values reported are adjusted for multiplicity. **: p<0.01, ***: p<0.005, ****: p<0.001.

Given the observed nsp1 targeting of a conserved site within the Rhileki and human 40S ribosomal subunit, we set out to understand the breadth of its inhibitory activity across a wide range of hosts known to be infected by SARS-CoV-2, including cats, dogs, minks, and deers^41–43^. We therefore extended the translation assay described in Figure 1A to other animal cell lines, including Crandell-Rees feline kidney epithelial cells (CRFK), canine fibroblasts cells (A72), American mink epithelial cells (Mv 1 Lu), Southern red muntjac deer fibroblast cells (Muntjac), and pig testis fibroblast cells (ST) (Fig S5). These mammals are susceptible to SARS-CoV-2 infection except pigs, which we included to assess nsp1 activity in a species non-permissive to SARS-CoV-2 replication^32–34,47^. Nsp1 mediated potent and concentration-dependent translation inhibition in CRFK, A72, Mv 1 Lu, Muntjac, and ST, whereas the nsp1 K164A/H165A double mutant did not (Fig 3, Fig S1). These results demonstrate that nsp1 inhibits protein translation in a wide range of mammalian hosts, concurring with the strict conservation of its binding site on the ribosome across mammals (Fig S3E).

## Discussion

Establishment of a successful viral infection depends on multiple factors, including viral entry, innate immune antagonism, and the ability to hijack cellular machineries for replication. For instance, the Nipah virus and Hendra virus V and W proteins, which result from frameshifting of P transcripts, inhibit interferon signaling responses with the V proteins also being a determinant of pathogenicity *in vivo*^65*–*71^. The lack of V and W proteins in the related Cedar virus renders it unable to mediate similar innate immune antagonism which has been proposed to contribute to the low pathogenicity of this virus^72^. Studies of non-structural proteins involved in innate immune antagonism are therefore key to understanding host species tropism, spillover, and pathogenicity.

Our results unveil the architecture of the *Rhinolophus lepidus* 40S ribosomal subunit, which is extraordinarily conserved with its human counterpart despite extensive evolutionary divergence between the Primate and Chiropteran orders. Indeed, SARS-CoV-2 nsp1 inhibits translation in two bat cell lines and cell lines from cats, dogs, minks, deers, and pigs by obstructing a highly conserved site in the mRNA entry channel, providing a molecular rationale for the broadly inhibitory nsp1 activity in several mammalian species. Nsp1 has also evolved to interfere with host mRNA in various ways such as prevention of host mRNA export from the nucleus^73^, induction of host mRNA cleavage^74^, and promoting evasion of direct Natural Killer cell cytotoxicity^75^. The multipronged role of nsp1 in immune evasion underscores the evolutionary arms race between viruses and hosts and the importance of nsp1 as a crucial factor in viral fitness for several coronaviruses^76–78^.

Nsp1 is one of the key players of the highly coordinated viral strategy to antagonize the host innate immune response by suppressing translation of antiviral genes such as interferons and NF-kB^79^. SARS-CoV-2-mediated innate immune antagonism also relies on several other non-structural proteins, such as ORF6 which blocks nuclear import and export^80^, rendering host cells incapable of responding to SARS-CoV-2 infection. As nsp1 efficiently inhibits translation in Rhileki and Tb 1 Lu cells, the downstream effects of innate immune suppression are expected to be similar between bats and humans due to nsp1 targeting a conserved pathway. Future studies will investigate the activity of other SARS-CoV-2 and sarbecovirus innate immune modulators in bats to further our understanding of the mechanisms underlying immune tolerance of pathogenic sarbecoviruses.

Numerous viruses hinder host protein translation to selectively promote production of viral proteins through various strategies. At the transcriptional level, a multitude of viruses encode proteins that disrupt RNA polymerase II^81–83^ whereas others block the nuclear mRNA export machinery^73,84^. Targeting cellular mRNAs for degradation^53,85^ or preventing them from being translated through direct interference with translation factors, such as the eIF4F complex and poly(A)-binding protein, are other examples of strategies employed by viruses^86,87^. Despite the variety of approaches evolved to interfere with host protein translation, obstruction of the 40S ribosomal subunit mRNA channel is unique to nsp1^88^. Some alpha-CoV nsp1s also interact with the 40S ribosomal subunit to suppress host translation^89^ through a presumably distinct mechanism of action due to their lack of CTD. Conversely, host proteins have evolved to perform a similar role to nsp1. For instance, SERPINE mRNA-binding protein 1 (SERBP1) and tumor suppressor Pdcd4 are cellular factors binding to the mRNA entry channel and obstructing it to regulate translation initiation^90,91^. These various pathways illustrate the plethora of molecular solutions that evolved to control protein translation in physiological and pathological processes.

The continued circulation and antigenic evolution of SARS-CoV-2 is associated with successive waves of infection globally, imposing considerable stress on public health infrastructures^92–96^. The ability of nsp1 to mediate translation inhibition in a broad spectrum of mammalian species shows that it is not a limiting factor for SARS-CoV-2 spillovers to other species and brings up the potential for further spread of SARS-CoV-2 into new hosts, as previously observed in white-tailed deers and farmed minks^42,97^. The conserved nsp1-binding site on the 40S ribosomal subunit along with the reduced nsp1 genetic drift among SARS-CoV-2 variants^98^, relative to the spike glycoprotein^94,96,99^, suggest the possibility of targeting this key virulence factor to develop next-generation COVID-19 countermeasures.

### Limitation of the study

Our study reveals the molecular basis of SARS-CoV-2 nsp1 CTD recognition of the Rhileki 40S ribosomal subunit but does not provide information about the effect of the nsp1 NTD on translation inhibition.

## Resource availability

### Lead contact

Further information and requests for resources and reagents should be directed to and will be fulfilled by the lead contact David Veesler (dveesler@uw.edu).

### Materials availability

Materials generated in this study will be made available after completion of a materials transfer agreement.

### Data and code availability

The cryo-EM model and maps have been deposited in the protein data bank and electron microscopy data bank with accession numbers 9NPX and EMD-49635 (Rhileki 40S body), 9NPY and EMD-49636 (Rhileki 40S head), and 9NPZ and EMD-49637 (Rhileki 40S consensus).

## STAR Methods

### Experimental Model and Study Participant Details

#### Cell Culture and Reagents

Cell lines used in this study were HEK293T (Female cell line, CRL-3216), Rhileki (Unsexed)^51^, Tb 1 Lu (Female cell line, CCL-88), CRFK (Female cell line, CCL-94), Muntjac (Male cell line, CCL-157), ST (Male cell line, CRL-1746), A72 (Female cell line, CRL-1542), and Mv 1 Lu (Male and female mixed cell line, CCL-64). HEK293T, Tb 1 Lu, A72, and CRFK were cultured in 10% FBS (Fisher Scientific-Cytiva), 1% penicillin-streptomycin (Thermo Fisher Scientific) in Dulbecco’s Modified Eagle Medium (DMEM) at 37°C, 5% CO_2_. Rhileki was cultured in 10% FBS, 1% penicillin-streptomycin, 1% sodium pyruvate (Thermo Fisher Scientific), and 1% non-essential amino acids (Thermo Fisher Scientific) in DMEM. ST and Mv 1 Lu were cultured in 10% FBS in Eagle’s Minimum Essential Medium (EMEM). Muntjac was cultured in 20% FBS in Hamster’s F-10 Nutrient Mix (Thermo Fisher Scientific).

### Method Details

#### Construct Design

pMCSG53 was used for cloning in *E. coli* DH10B cells, and for recombinant protein expression in *E. coli* BL21 (DE3) cells. SARS-CoV-2 Nsp1 full length (NC_045512.2) was cloned into pMCSG53 with a 6xHis and TEV (Tobacco Etch Virus) tag inserted before the N terminal domain for purification and cleavage.

eGFP transcript was obtained from Genscript including a 5’ cap, synthetic nonviral 5’ UTR (sequence: AAATAAGAGAGAAAAGAAGAGTAAGAAGAAATATAAGA), and 100 nt polyA tail. Nsp1 WT and mutant transcripts were obtained from Trilink with a 5’ cap, AGG initiation codon followed by genomic SARS-CoV-2 5’ UTR, 3XFLAG tag, and 80 nt polyA tail. All transcripts include N1-methyl pseudouridines in substitute of uridines.

#### Bacterial Expression and Purification of Recombinant nsp1

Nsp1 was transformed and expressed in Escherichia coli BL21 (DE3) cells. Cells were grown at 37°C until the OD_600_ reached 0.6, when the cells were then induced by 1.0 mM isopropyl β-D-1-thiogalactopyranoside (IPTG) and shifted to incubation at 18°C for 18 hours in Luria’s Broth in the presence of ampicillin. Cells were harvested via centrifugation at 7000xg for 30 min, resuspended in lysis buffer [20 mM HEPES-NaOH pH 8.0, 500 mM NaCl, 5 mM MgCl2, 10 mM imidazole, 10% glycerol, 0.5 mM TCEP], and lysed using a C5 Emulsiflex. The lysate was clarified via centrifugation at 11,000xg for 40 min and filtered through a 0.45 μm membrane before being applied to a HisTrap HP column (Cytiva). Proteins were washed in lysis buffer supplemented with 20 mM imidazole before being eluted in lysis buffer supplemented with 300 mM imidazole. TEV cleavage was performed immediately after in TEV reaction buffer [50 mM Tris-HCl pH 8.0, 0.5 mM EDTA, 1mM DTT, 5% glycerol] with TEV protease (1 uL per 30 ug protein) and incubated at 30°C for 1 hour. SDS-PAGE gel was run to analyze cleavage products. Concentration of Nsp1 was performed in Amicon Ultra-15 Centrifugal Filter Concentrators with a 10 kDa molecular weight cutoff. Size exclusion chromatography (HiLoad Superdex 75, GE healthcare) was performed at 4°C in storage buffer [20 mM HEPES-NaOH pH 7.5, 500 mM NaCl, 5 mM MgCl2, 10% glycerol, 1 mM TCEP]. Purity of the proteins were analyzed by SDS-PAGE after each step.

#### Bacterial Expression and Purification of Recombinant eIF1

Human eIF1 was purified as described previously^64,100^. Briefly, recombinant human eIF1 was expressed in *E. coli*, cleaved using recombinant TEV protease, and purified via ion-exchange chromatography.

#### Purification of 40S ribosomal subunits

40S ribosomal subunits were purified from Rhileki cells following previously established protocols (Khatter et al., 2014) Rhileki cells were trypsinized to detach from cell culture flasks and pelleted via centrifugation at 1200 rpm for 5 min. The cell pellet was resuspended in lysis buffer [30 mM Tris-HCl pH 7.5, 300 mM NaCl, 30 mM MgCl2, 2% (v/v) Triton-X 100, 4 mM DTT] supplemented with RNAsin (Promega) and Halt protease inhibitor (Thermo) and incubated on ice for 10 min. Following brief centrifugation at 10,000 rpm for 10 min at 4°C, the lysate was loaded on a 30% sucrose cushion [20 mM Tris-HCl pH 7.5, 500 mM KCl, 30% (v/v) sucrose, 10 mM MgCl2, 0.1 mM EDTA pH 8.0, and 2 mM DTT] and pelleted via ultracentrifugation for 18 hours at 30,000 rpm in a Beckman Ti60 rotor at 4°C. Ribosomes were then resuspended in resuspension buffer [20 mM Tris-HCl pH 7.5, 500 mM KCl, 7.5% v/v sucrose, 2 mM MgCl2, 75 mM NH4Cl, 2mM puromycin, 2 mM DTT] supplemented with RNAsin and Halt protease inhibitor and incubated at 4°C for 1 hour, followed by 37°C for 1.5 hours. The resuspended ribosomes were then loaded on a 11% to 25% sucrose gradient [20 mM Tris-HCl pH 7.5, 500 mM KCl, 11%–25% (v/v) sucrose, 6 mM MgCl2] using a Beckman SW 32 Ti rotor at 17,000 rpm for 16 hours at 4°C. Sucrose gradient was prepared using the Biocomp gradient master. Fractions were collected by the Biocomp piston fractionator and fractions containing 40S subunits were combined, and concentrated using the Amicon Ultra-15 Centrifugal Filter Concentrators with a 100 kDa cutoff while being buffer exchanged into storage buffer [30 mM HEPES-KOH pH 7.4, 100 mM KOAc, 5 mM Mg(OAc2), 6% (v/v) sucrose, 2mM DTT]. After negative stain and 1% bleach agarose gels were used to confirm sample purity, small aliquots were flash frozen in liquid nitrogen and stored at −80°C.

#### Transfection Assay and Western Blot Analysis of GFP Expression

Day before transfections, cells were seeded in a black 96-well glass bottom plate (Cellvis). The next day, 200 ng eGFP mRNA alone or eGFP with 0.01 ng, 0.1 ng, or 1 ng nsp1/nsp1 mutant were mixed with Lipofectamine 2000 in a 1ug:3uL ratio of mRNA to Lipofectamine and added to each well for six technical replicates. The cells were incubated with the transfection mixture for 16 hours in a 37°C, 5% CO_2_ incubator. Right before measurements, media was removed from the cells and replaced with pre-warmed FluoroBrite DMEM. Fluorescence was detected and quantified using the Biomek Cytation 5 with a GFP filter. The 16-bit raw images were processed in ImageJ to adjust the color display to green and a maximum value of 37870 was set for all images.

For GFP expression western blot analysis, after GFP quantification, cells were washed with ice-cold 1X phosphate buffered saline (PBS, pH 7.4) before adding 10 uL RIPA Lysis and Extraction buffer (Thermo) supplemented with SIGMAFAST^™^ Protease Inhibitor Cocktail Tablets (Sigma-Aldrich) to each well and incubated for 5 min. The cell lysate was collected and centrifuged for 15 min at 14,000xg at 4°C. The supernatant was collected and combined with 4X SDS-PAGE Sample Loading Buffer and boiled at 95°C for 5 min. 8 uL of each transfection sample (along with non-transfected negative control) was run through a 4%–20% gradient Tris-Glycine gel (BioRad) and transferred to a PVDF membrane using the mixed molecular weight protocol of the Trans-Blot Turbo System (BioRad). The membrane was blocked with 5% milk in TBS-T (20 mM Tris-HCl pH 8.0, 150 mM NaCl) supplemented with 0.05% Tween-20 at room temperature and with agitation. After 1 h, the GFP monoclonal antibody (1:3000 dilution, GF28R, Thermo Fisher) and incubated for 1h at room temperature with agitation. The membrane was washed three times with TBS-T and an IRDye 680 RD Goat anti-Mouse IgG secondary antibody (1:20,000 dilution, LI-COR, 926–68070) was added and incubated for 1 h at room temperature. The membrane was washed three times with TBS-T after which a LI-COR processor was used to develop the western blot. Membranes in figures were cropped to show area of interest.

#### Transfection Assay and Western Blot Analysis of Nsp1 Expression

Day before transfections, cells were seeded in a 12-well cell culture plate (Genesee Scientific) and incubated overnight. The next day, 400 ng, 200 ng, and 100 ng of nsp1 and nsp1 KH mutant were transfected into each well with Lipofectamine 2000 in a 1ug:3uL ratio of mRNA to Lipofectamine and incubated overnight. After 16 hours, cells were washed twice with ice-cold 1X phosphate buffered saline (PBS, pH 7.4) before adding 50 uL RIPA Lysis and Extraction buffer (Thermo) supplemented with SIGMAFAST^™^ Protease Inhibitor Cocktail Tablets (Sigma-Aldrich) for 5 min. The cell lysate was collected and centrifuged for 15 min at 14,000xg at 4°C. The supernatant was collected and combined with 4X SDS-PAGE Sample Loading Buffer and boiled at 95°C for 5 min.

For nsp1 expression western blot analysis, 8 uL of each transfection sample (along with non-transfected negative control) except for the Rhileki cell lysates for which 30 uL was used instead, was run through a 4%–20% gradient Tris-Glycine gel (BioRad) and transferred to a PVDF membrane using the mixed molecular weight protocol of the Trans-Blot Turbo System (BioRad). The membrane was blocked with 5% milk in TBS-T (20 mM Tris-HCl pH 8.0, 150 mM NaCl) supplemented with 0.05% Tween-20 at room temperature and with agitation. After 1 h, the nsp1 polyclonal antibody (1:1000 dilution, SARS-CoV-2 Nsp1 Antibody, Thermo Fisher PA5–11694) and incubated overnight at 4°C with agitation. The next day, the membrane was washed three times with TBS-T and an IRDye 680RD Goat anti-Rabbit IgG secondary antibody (1:20,000 dilution, LI-COR, 926–68073) was added and incubated for 1 h at room temperature. The membrane was washed three times with TBS-T after which a LI-COR processor was used to develop the western blot.

#### PCR amplification of 18S ribosomal RNA and eS10

One day before extraction, Rhileki cells were plated on two 100 mm cell culture plates (Corning) and incubated overnight in 37°C, 5% CO_2_. The next day, cells were washed with ice-cold 1X PBS and total RNA was extracted from Rhileki cells using the Quick-RNA Miniprep Kit (Zymo Research) following the recommended protocol. Extracted RNA was quantified and stored at −80°C. For PCR amplification of ribosomal protein S10 and 18S rRNA, primers were designed based on the *Rhinolophus ferrumequinum* sequences (GCF_004115265.2) with forward primer ACCTGGTTGATCCTGCCAGTAG and reverse primer TAATGATCCTTCCGCAGGTTCACC for 18S, and forward primer CCGGGACGYMGAAGTCGTAA and reverse primer TGCAAAGAYGGGCACACAAG for eS10. cDNA was subsequently synthesized using the Lunascript RT Supermix (NEB) with the recommended protocol. PCR reactions were performed with KAPA HiFi HotStart ReadyMix (Roche) and CloneAmp HiFi PCR Premix (Takara Bio). PCR products were run on 1% Tris acetate-EDTA (TAE) agarose gels and extracted using the Zymoclean Gel DNA Recovery Kit (Zymo). Products were sent for sequencing for confirmation of the correct amplicon.

#### Transcriptome sequencing sample preparation and data analysis

Total RNA from Rhileki cells was extracted as described above. Sequencing libraries were prepared using the TruSeq Stranded mRNA kit (Illumina) and sequenced on a 2 × 150 bp run on an Illumina NovaseqX+ by Psomagen. A total of 116,395,242 sequencing reads were obtained for the sample. The sequencing reads were quality and adapter trimmed using Trimmomatic v0.39 and aligned to the homologous ribosomal transcripts of *R. ferrumequinum* (RefSeq accession number: GCF_004115265.2) using Geneious Prime. The sequence alignments were visualized in Geneious Prime and the final sequences of the *R. lepidus* ribosomal transcripts were manually called. Sequencing reads are available under the NCBI Bioproject PRJNA1190498 and ribosomal coding sequences are included in supplementary materials.

#### Cryo-EM Sample Preparation, Data Collection, and Data Processing

Nsp1 bound Rhileki 40S ribosomal subunit complex was prepared by mixing a 1:5:5 molar ratio of 40S ribosomal subunit:nsp1:eIF1 followed by a 15 minute incubation at room temperature and kept on ice for the remaining time until grids were frozen. 3 uL of 80 nM complex was applied onto fresh glow discharged R 2/2 UltrAuFoil grids prior to plunge freezing using a vitrobot MarkIV (ThermoFisher Scientific) with a blot force of −1, 6 sec blot time, and 40 sec waiting time at 100% humidity and 22°C. The data was acquired using an FEI Titan Krios transmission electron microscope operated at 300 kV and equipped with a Gatan K3 direct detector and Gatan Quantum GIF energy filter, operated in zero-loss mode with a slit of 20 eV. Automated data collection was carried out using SerialEM^101^ at a nominal magnification of 105,000x with a pixel size of 0.829 Å. A total of 14,216 micrographs were collected with a defocus range between −0.6 and −1.6 μm with stage tilt angle of 0° and 30°. Movie frame alignment, estimation of the microscope contrast-transfer function parameters, particle picking, and extraction were carried out using cryoSPARC Live^102^, and all downstream processing steps were done in cryoSPARC^103^. Particles were extracted with a box size of 432 pixels and downsampled by a factor of 6. One round of reference-free 2D classification was performed to select well-defined particle images. Initial model generation was performed using ab-initio reconstruction and the resulting map was used for non-uniform refinement. The resulting map was used to re-extract 1,205,667 particles with extraction box size of 512 pixels cropped to 384 pixels. The re-extracted particles were used for subsequent non-uniform refinement with per-particle defocus and global CTF refinement. This was followed by reference-based motion correction and subsequent non-uniform refinement with per-particle defocus, global CTF refinement, and Ewald sphere negative correction to obtain the consensus map at 2.1 Å (EMD-49637). Masks for the 40S head and body were created in UCSF ChimeraX^104^ and low pass filtered to 20Å in cryoSPARC. Local refinement for the head and body regions were conducted separately using the same consensus map in order to yield final reconstructions of the 40S head and body maps at 2.1Å (EMD-49636 and EMD-49635. Reported resolutions are based on the gold-standard Fourier shell correlation (FSC) of 0.143 criterion. See also Table S1 and Fig S4.

#### Model Building

Initial model building used ModelAngelo^105^ with the Rhileki 40S - nsp1 consensus map and sequences for nsp1, eIF1, and the 40S ribosomal subunit (Table S1). The structure was then manually rebuilt and extended in the locally refined head and body maps using Coot^106^ and refined using Phenix^107^, ISOLDE^108^ and Rosetta^109^. Human 40S structures were used to assist the process (PDB: 8PPK)^39^ (Table S1). Validation used Molprobity^110^ and Phenix. Modified residues and nucleotides were included according to density and using human 80S ribosome (PDB: 8QOI)^111^ and human 40S ribosomal subunit (PDB: 8PPK) as guides. Pseudouridines were added to the model when justified by the presence of a water molecule next to the N1 position of the base^111^. Structures are deposited in the PDB with accession codes 9NPZ, 9NPY, and 9NPX for the consensus (excluding head region except RPS3), body, and head structures respectively.

### Quantification and Statistical Analysis

Transfection assays were conducted 6 times with 6 biological repeats. Data were presented by means ± SD as indicated in the figure legends. One-way ANOVA and Dunnett’s T3 multiple comparison tests were conducted for all statistical analyses using GraphPad Prism 8 unless specified otherwise. *P* < 0.05 was considered significant. *P < 0.05,**P < 0.01, ***P < 0.005, and ****P < 0.001.

## Supplementary Material

TableS1

FigureS1

FigureS2

FigureS3

FigureS4

FigureS5

DocumentS1. FiguresS1-S5

Supplemental Information

Document S1. Supplemental figures S1–S5.

Table S1, related to Figures 2 and S4. Cryo-EM data collection and statistics, model content and sequences, 40S, rRNA, and 60S sequences, and ribosome sequence percentage identities.

## Figures and Tables

**Key resources table:** 

REAGENT or RESOURCE	SOURCE	IDENTIFIER
Antibodies		
SARS-CoV-2 NSP1 Polyclonal Antibody	Thermo Fisher	Cat# PA5-116941; RRID: AB_2901571
GFP Monoclonal Antibody (GF28R)	Thermo Fisher	Cat # MA5-15256; RRID: AB_10979281
IRDye^®^ 680RD Goat anti-Rabbit IgG Secondary Antibody	LICORbio	Cat # 926-68071; RRID: AB_2721181
IRDye^®^ 680RD Donkey anti-Mouse IgG Secondary Antibody	LICORbio	Cat # 926-68072; RRID: AB_2814912
Bacterial and virus strains		
DH10B	Thermo Fisher	EC0113
BL21(DE3)	Thermo Fisher	EC0114
Chemicals, peptides, and recombinant proteins		
Tris-HCI	Thermo Fisher	15567027
EDTA	Thermo Fisher	15-575-020
Agarose	Thermo Fisher	16500500
TBE	Thermo Fisher	AM9865
RNasin^®^ Ribonuclease Inhibitor	Promega	N2515
Sucrose	Thermo Fisher	J65148.36
DTT	Thermo Fisher	R0861
KCl	Thermo Fisher	AM9640G
MgCl_2_	Thermo Fisher	AM9530G
NaCl	Thermo Fisher	AM9760G
Magnesium acetate tetrahydrate	Millipore Sigma	M5661-50G
HEPES	Thermo Fisher	J16926.22
Potassium acetate	Thermo Fisher	AM9610
Halt^™^ Protease Inhibitor Cocktail	Thermo Fisher	78429
S.O.C. medium	Thermo Fisher	15544034
DMEM	Thermo Fisher	11-965-126
Eagle’s Minimum Essential Medium	ATCC	30-2003
Sodium pyruvate	Thermo Fisher	11360070
Non-essential amino acids	Thermo Fisher	11140050
Fetal Bovine Serum	Cytiva	SH3039603HI
Penicillin-Streptomycin	Thermo Fisher	15140163
PBS	Thermo Fisher	10010031
Ham's F-10 Nutrient Mix	Thermo Fisher	11550043
Lipofectamine 2000	Thermo Fisher	11668500
Trypsin-EDTA	Thermo Fisher	25200114
Isopropyl β-D-1-thiogalactopyranoside	Millipore Sigma	I5502-1G
TCEP	Millipore sigma	C4706-10G
Imidazole	Millipore Sigma	56749
Triton X-100	Millipore Sigma	X100
Glycerol	Thermo Fisher	A16205.AP
Tween 20	Thermo Fisher	BP337-500
Instant Nonfat Dry Milk	Nestle	12428935
SYBR Safe	Thermo Fisher	S33102
Critical commercial assays		
Quick-RNA Miniprep Kit	Zymo Research	R1054
LunaScript^®^ RT SuperMix Kit	New England Biolabs	E3010S
Zymoclean Gel DNA Recovery Kit	Zymo Research	D4002
KAPA HiFi HotStart ReadyMix	Roche	50-196-5299
CloneAmp^™^ HiFi PCR Premix	Takara Bio	639298
Deposited data		
Rhinolophus lepidus 40S ribosomal subunit bound to SARS-CoV-2 nsp1 (body)	PDB	9NPX
Rhinolophus lepidus 40S ribosomal subunit bound to SARS-CoV-2 nsp1 (head)	PDB	9NPY
Rhinolophus lepidus 40S ribosomal subunit bound to SARS-CoV-2 nsp1	PDB	9NPZ
Electron density map (consensus)	EMDB	
Electron density map (focus refined 40S head)	EMDB	
Electron density map (focus refined 40S body)	EMDB	
Experimental models: Cell lines		
Tb 1 Lu	ATCC	CCL-88
ST	ATCC	CRL-1746
Mv 1 Lu	ATCC	CCL-64
Indian Muntjac	ATCC	CCL-157
CRFK	ATCC	CCL-94
A72	ATCC	CRL-1542
HEK293T	ATCC	CRL-3216
Rhileki	Auerswald, H. et al.	N/A
Oligonucleotides		
Rhinolophus lepidus 18S rRNA Forward (ACCTGGTTGATCCTGCCAGTAG)	IDT	N/A
Rhinolophus lepidus 18S rRNA Reverse (TAATGATCCTTCCGCAGGTTCACC)	IDT	N/A
Rhinolophus lepidus eS10 Forward (CCGGGACGYMGAAGTCGTAA)	IDT	N/A
Rhinolophus lepidus eS10 Reverse (TGCAAAGAYGGGCACACAAG)	IDT	N/A
Recombinant DNA		
SARS-CoV-2 nsp1	BEI?	
Software and algorithms		
Adobe Illustrator	Adobe	https://www.adobe.com/products/illustrator.html
ImageJ	NIH	https://imagej.net/ij/download.html
cryoSPARC v4.4.0	Punjani et al.	https://cryosparc.com
Prism 10	Graphpad	https://www.graphpad.com/scientific-
Coot	Emsley et al.	https://www2.mrc-lmb.cam.ac.uk/personal/pemsley/coot/
ChimeraX	Pettersen et al.	https://www.cgl.ucsf.edu/chimerax/
Phenix	Liebschner, D. et al.	https://phenix-online.org/
Other		
PVDF membrane	Bio-Rad	1704156
RIPA Lysis and Extraction Buffer	Thermo Fisher	89901
InstantBlue^®^ Coomassie Protein Stain	Abcam	ab119211
UltrAuFoil	Electron Microscopy Sciences	Q250AR2A
