# Supplementary figures and images for: SARS-CoV-2 nsp1 mediates broad inhibition of translation in mammals
Source: Cell Rep. Author manuscript; Available in PMC 2026 Jun 8. (PMC13245623; doi:10.1016/j.celrep.2025.115696)

A

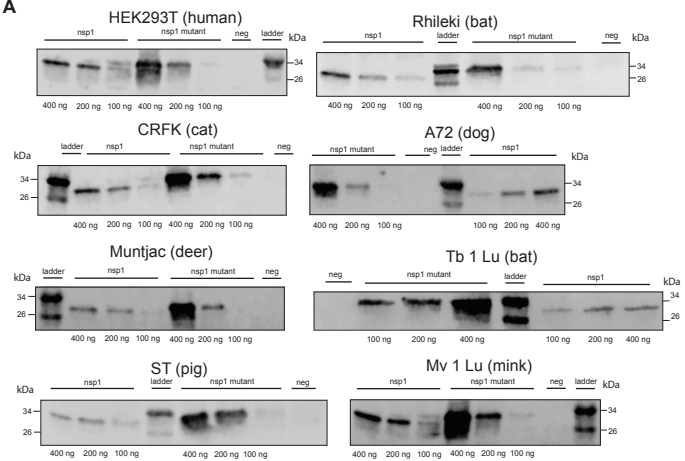

B

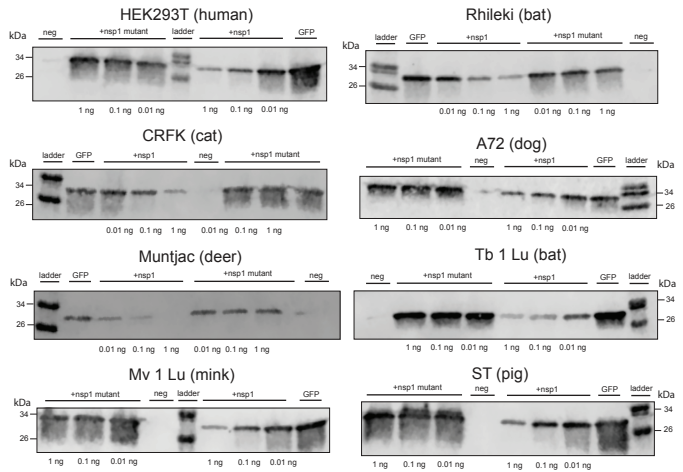

Supplement: FigureS1 [file NIHMS2171571-supplement-FigureS1.pdf]

**A**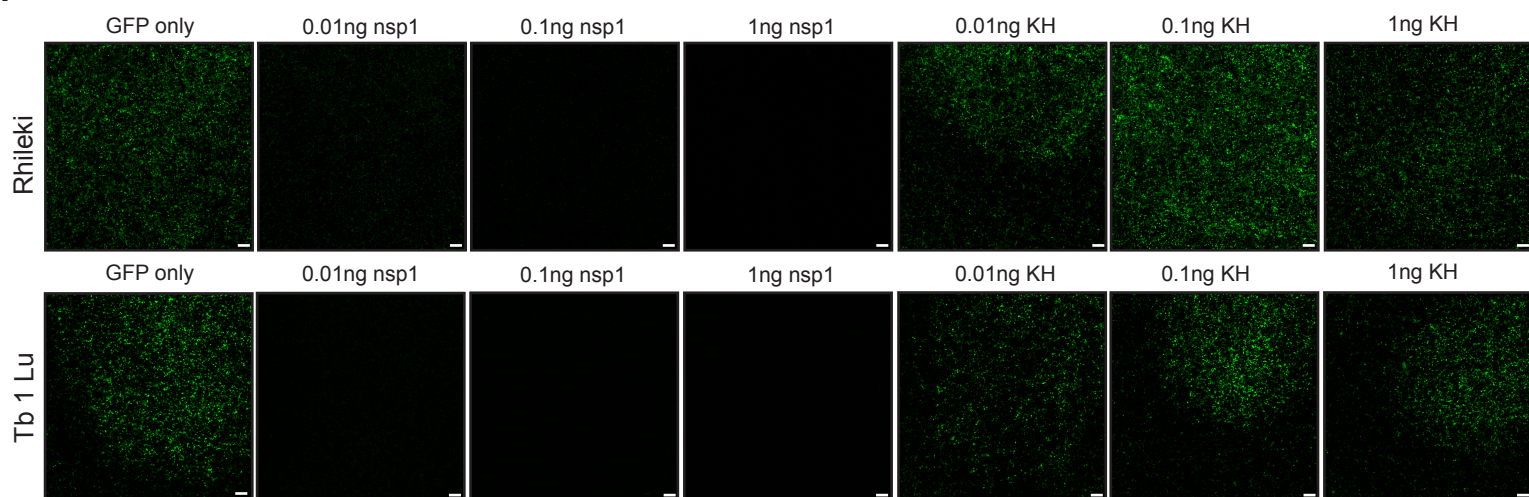**B**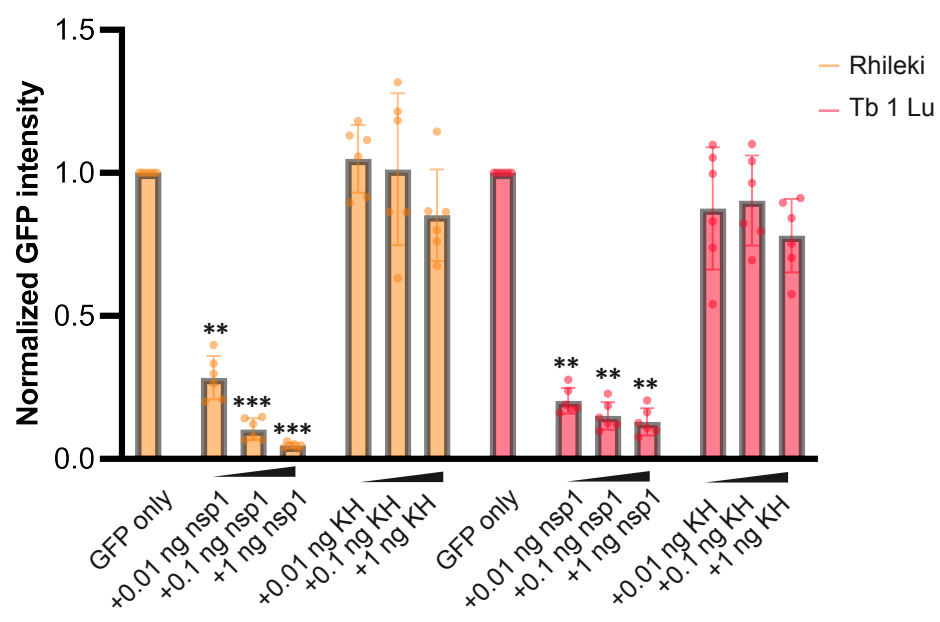

Supplement: FigureS2 [file NIHMS2171571-supplement-FigureS2.pdf]

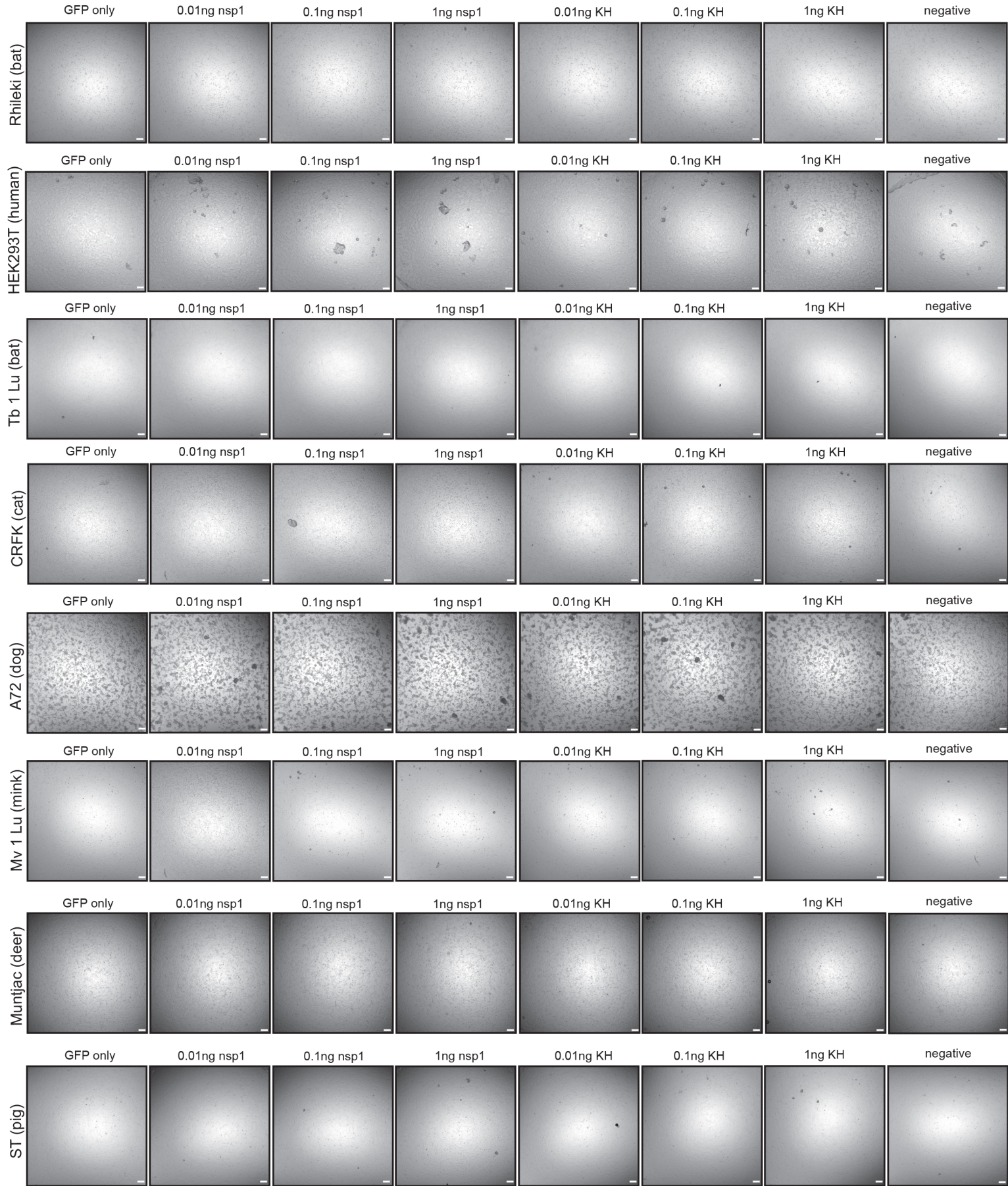

Supplement: FigureS5 [file NIHMS2171571-supplement-FigureS5.pdf]
