# Supplementary material for: SARS-CoV-2 nsp1 mediates broad inhibition of translation in mammals
Source: Cell Rep. Author manuscript; Available in PMC 2026 Jun 8. (PMC13245623; doi:10.1016/j.celrep.2025.115696)
Supplement: FigureS4 [file NIHMS2171571-supplement-FigureS4.pdf]

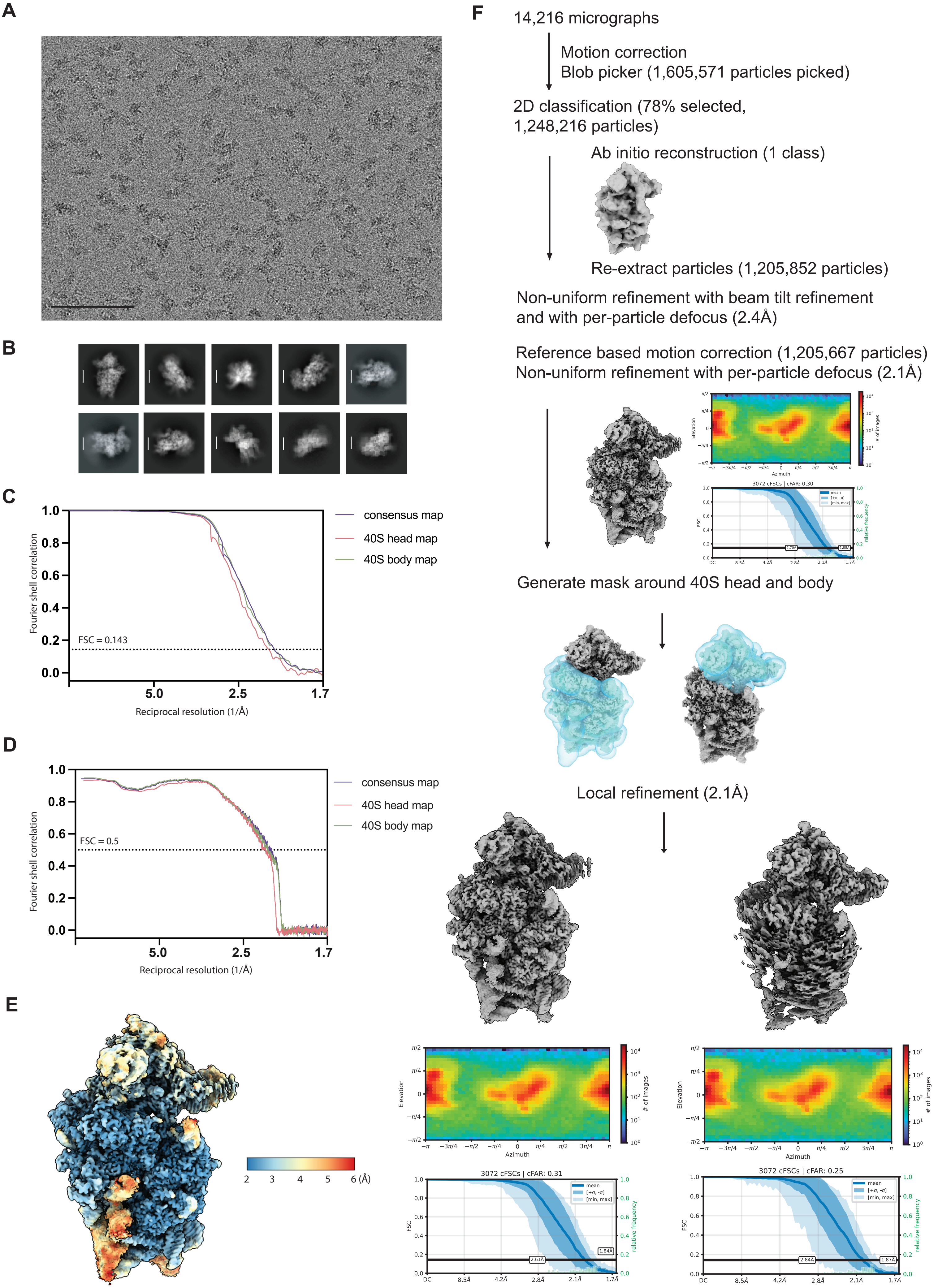
A
B
C
1.0
0.8
0.6
0.4
0.2
0.0
Fourier shell correlation
FSC = 0.143
5.0
2.5
1.7
Reciprocal resolution (1/Å)
consensus map
40S head map
40S body map
D
FSC = 0.5
E
2 3 4 5 6 (Å)
F
14,216 micrographs
Motion correction
Blob picker (1,605,571 particles picked)
2D classification (78% selected,
1,248,216 particles)
Ab initio reconstruction (1 class)
Re-extract particles (1,205,852 particles)
Non-uniform refinement with beam tilt refinement
and with per-particle defocus (2.4Å)
Reference based motion correction (1,205,667 particles)
Non-uniform refinement with per-particle defocus (2.1Å)
3072 cFSCs | cFAR: 0.30
Generate mask around 40S head and body
Local refinement (2.1Å)
Elevation
Azimuth
# of images
3072 cFSCs | cFAR: 0.31
3072 cFSCs | cFAR: 0.25
FSC
relative frequency
mean
[+σ, -σ]
[min, max]
2.61Å
1.84Å
2.84Å
1.87Å
DC
8.5Å
4.2Å
2.8Å
2.1Å
1.7Å
