# Supplementary material for: SARS-CoV-2 nsp1 mediates broad inhibition of translation in mammals
Source: Cell Rep. Author manuscript; Available in PMC 2026 Jun 8. (PMC13245623; doi:10.1016/j.celrep.2025.115696)
Supplement: DocumentS1. FiguresS1-S5 [file NIHMS2171571-supplement-DocumentS1__FiguresS1-S5.pdf]

Supplementary Figure S1

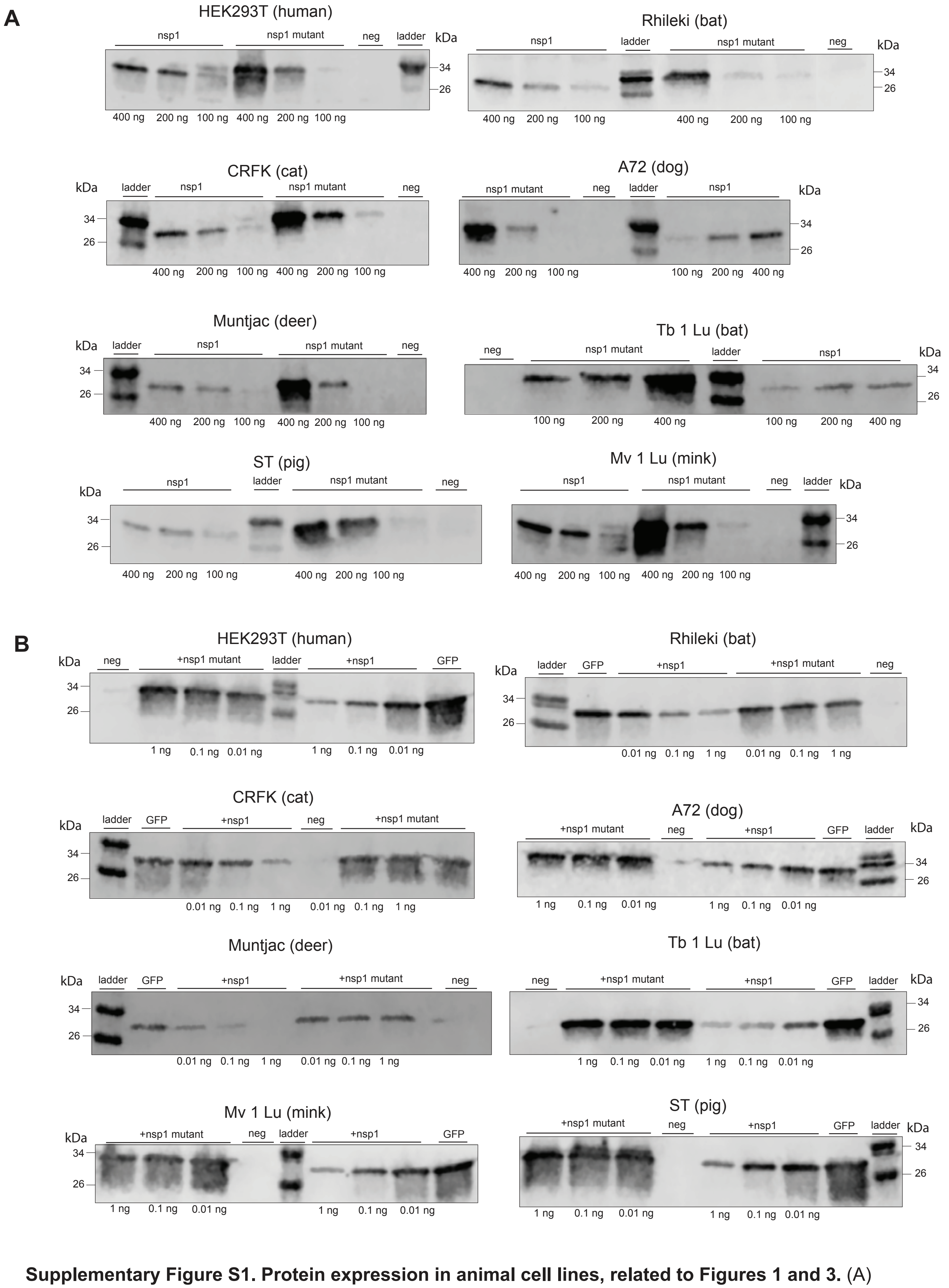

Supplementary Figure S1. Protein expression in animal cell lines, related to Figures 1 and 3. (A) Western blot analysis of nsp1 and nsp1 K164A/H165A mutant expression using anti-nsp1 polyclonal antibody (Thermo) in seven different animal cell lines with transfection amounts listed below the images. Transfection assays for these western blots were performed in 12-well plates with increased amounts of mRNA to enable detection of expression (which could not be detected in a 96-well format with much lower amounts of mRNA transfected as used in Figures 1 and 3). (B) Western blot analysis of GFP expression in the presence or absence of the wildtype or the K164A/H165A mutant nsp1 shown in Figures 1 and 3 using anti-GFP monoclonal antibody GF28R (Thermo). Neg: negative control.

Supplementary Figure S2

A

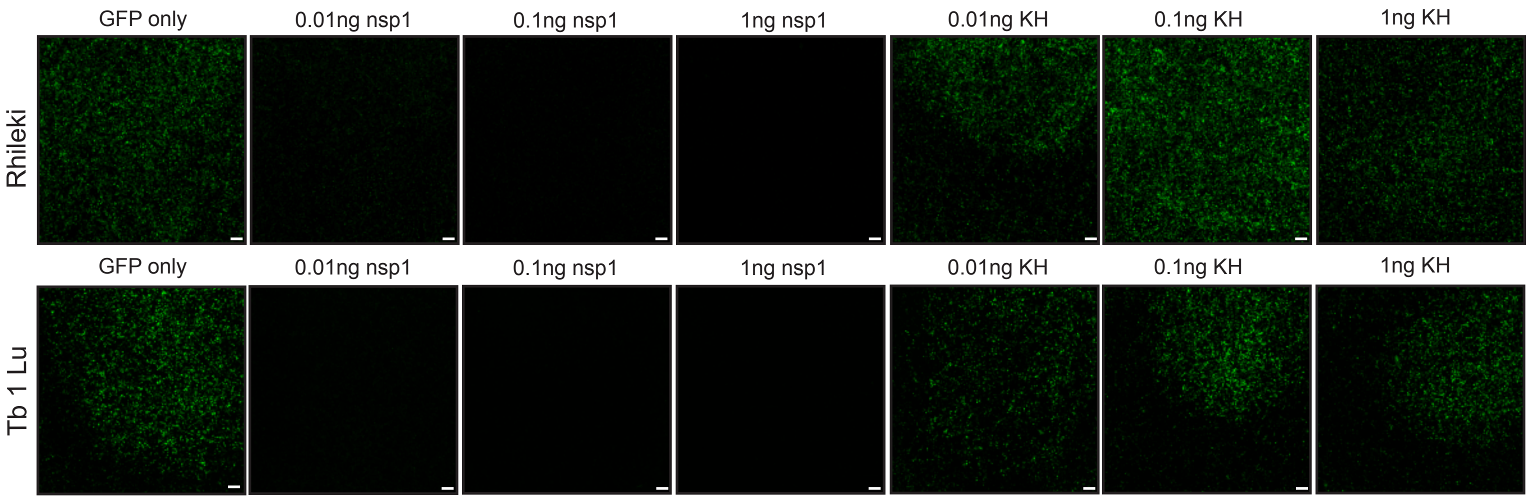

B

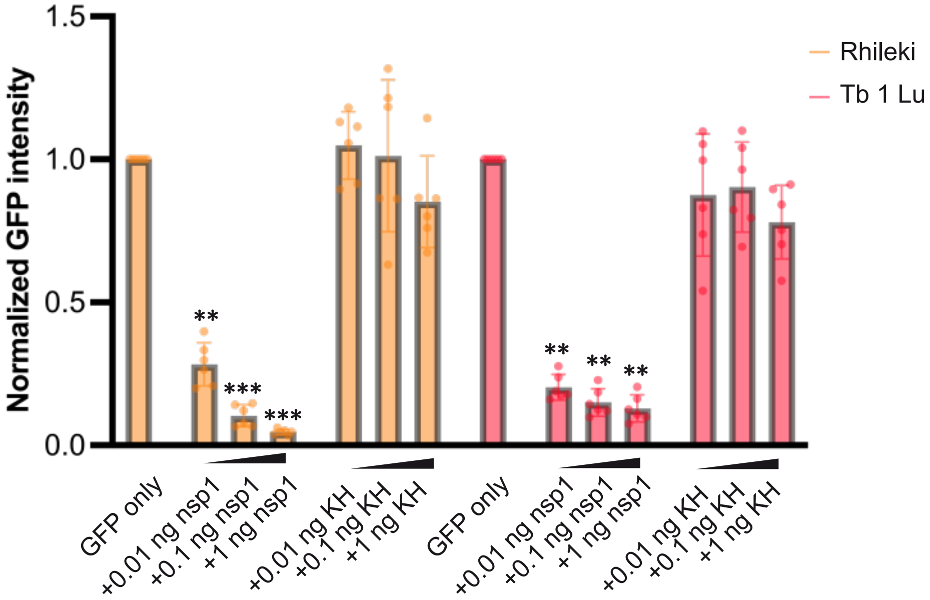

Supplementary Figure S2. SARS-CoV-2 nsp1 inhibits translation in two bat cell lines, related to Figure 1. (A) Live-cell fluorescence imaging 16h post transfection of GFP expression in the presence (or absence) of varying amounts of wildtype or K164A/H165A nsp1 mutant (KH) mRNA using Rhinolophus lepidus kidney epithelial (Rhileki) cells and Tadarida brasiliensis lung epithelial-like (Tb 1 Lu) cells. Scale bar: 100 μm. See Fig S1 for nsp1 and GFP expression levels. (B) Quantification of the nsp1-mediated dose-dependent inhibition of translation 16h post transfection based on normalized GFP fluorescence intensity across the entire field of view. Bars represent the mean of six biological replicates shown as individual data points with error bars showing standard deviation. Each biological replicate is a mean of six technical replicates. Transfections with wildtype and mutant nsp1 mRNAs were compared to the GFP-only control using one-way ANOVA and follow-up Dunnet's T3 multiple comparisons tests. P-values reported are adjusted for multiplicity. **: $p<0.01$, ***: $p<0.005$.

Supplementary Figure S3

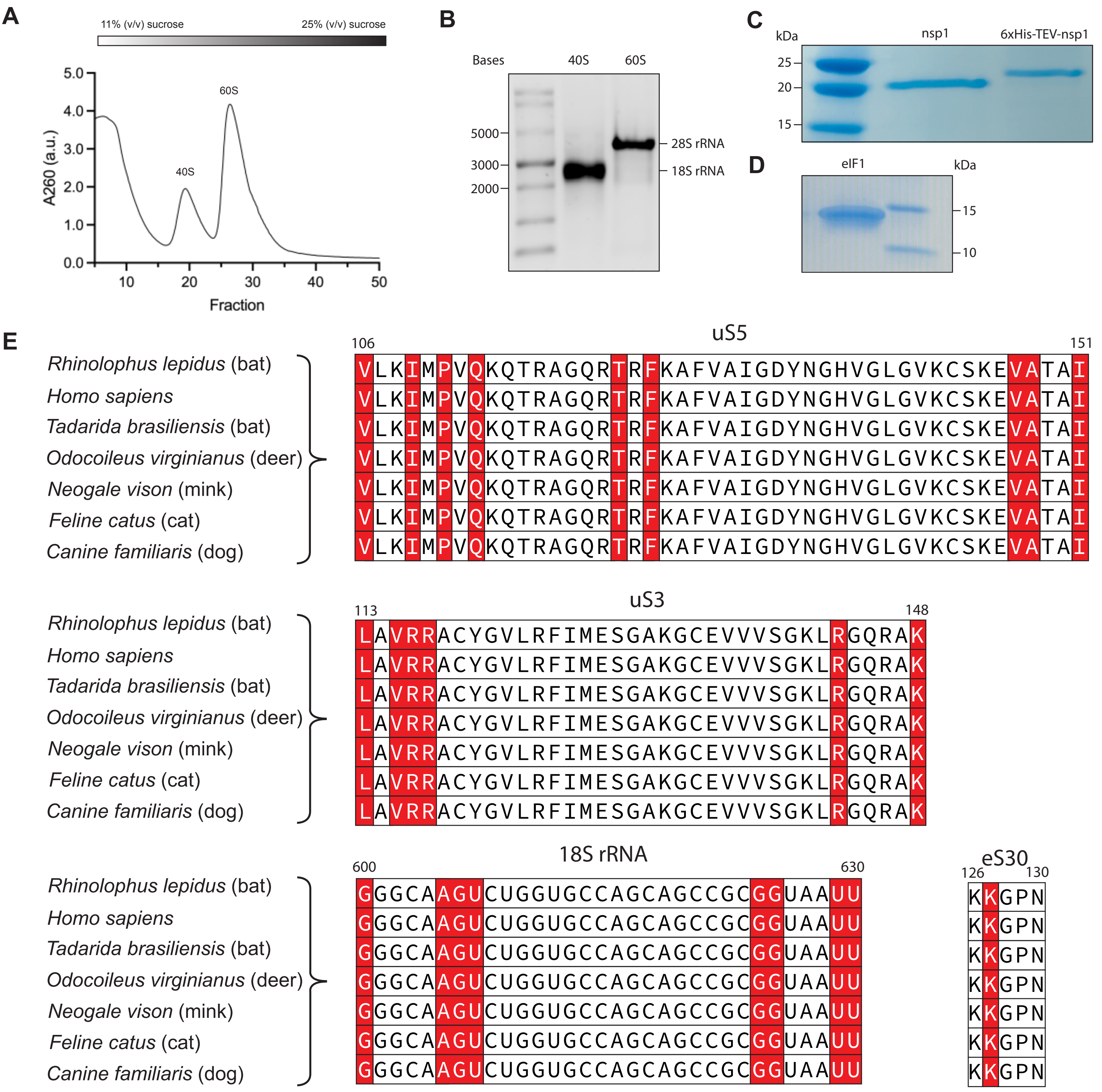

Supplementary Figure S3. Purification of bat Rhileki ribosome, SARS-CoV-2 nsp1, human eIF1, and consensus sequence of mammalian ribosomes, related to Figure 2.
(A) Isolation of the Rhileki ribosomal 40S and 60S subunits using a 11-25% (v/v) sucrose density gradient. The purification process was followed using absorbance at 260 nm (A260) of each fraction. (B) 1% bleach agarose gel showing separation of the 40S and 60S ribosomal subunits through 18S and 28S rRNA respectively. An RNA ladder was used to deduce separation of the 18S and 28S bands. (C) SDS-PAGE gel of recombinant SARS-CoV-2 nsp1 with or without the 6xHis-TEV tag. (D) SDS-PAGE gel of recombinant human eukaryotic initiation factor 1 (eIF1). TEV: Tobacco Etch Virus protease cleavage site. (E) Sequences of mammalian ribosomal proteins and rRNAs near nsp1-interacting residues (boxed in red). Residue numbering corresponds to R. lepidus.

Supplementary Figure S4

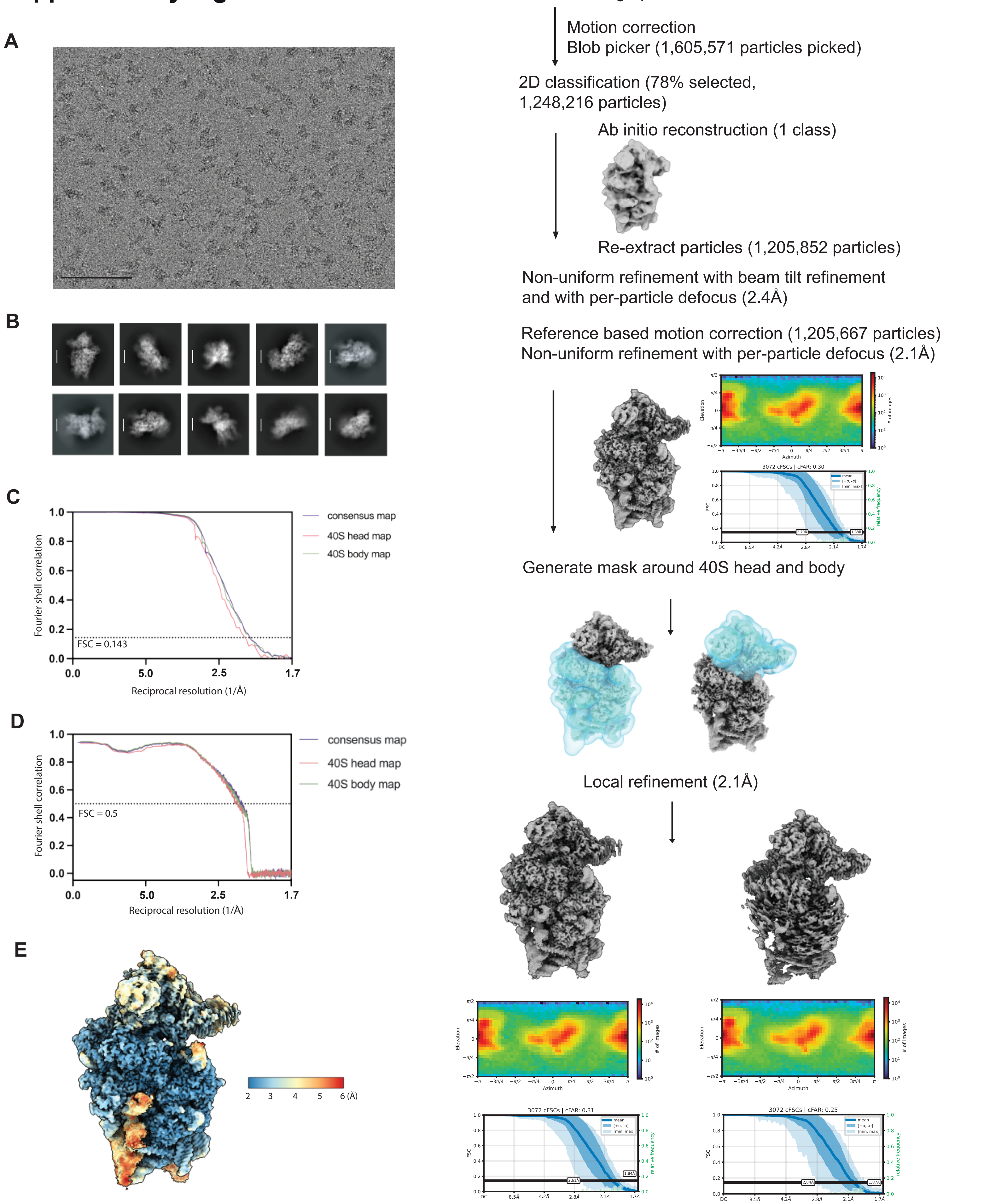

Supplementary Figure S4. Cryo-EM workflow of SARS-CoV-2 nsp1 bound 40S Rhileki ribosomal subunit structure, related to Figure 2. (A) Representative electron micrograph and (B) 2D class averages. Scale bars of the micrograph and class averages are 100 nm and 100Å, respectively. (C-D) Gold-standard fourier shell correlation (FSC, C) and model-map FSC (D) curves for the consensus map (purple) and the locally refined maps the 40S head (pink) and body (green). (E) Local resolution plotted on the unsharpened map of the consensus refinement. (F) Data processing flowchart for the reconstruction of the consensus map and the subsequent locally refined maps. Angular distribution plots with all the particles contributing to the final maps and three-dimensional FSC curves are shown for each of the three reconstructions.

Supplementary Figure S5

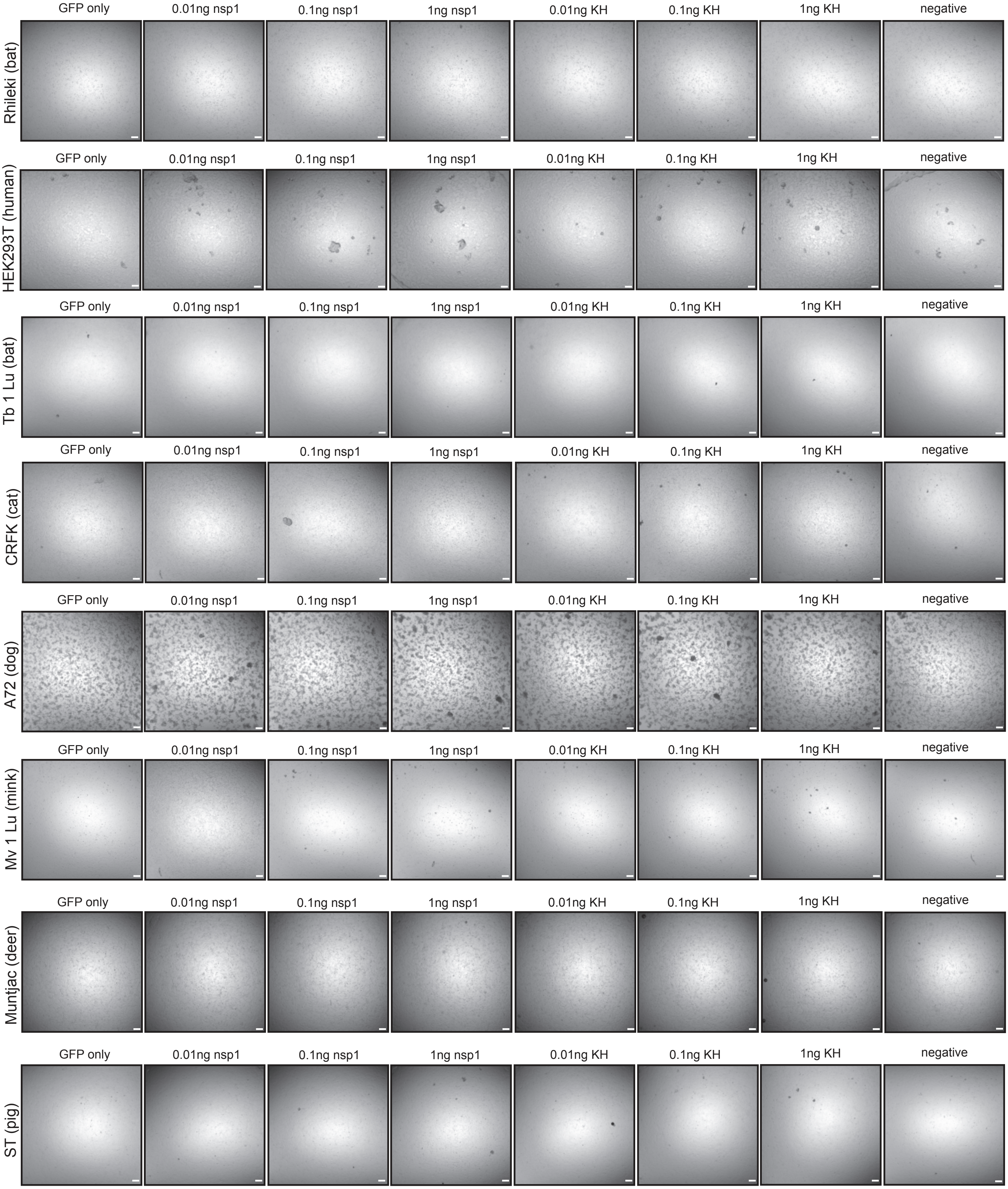

Supplementary Figure S5. Bright-field images of cell lines used for transfections, related to Figures 1 and 3. Corresponding bright-field images of fluorescence images. KH: nsp1 K164A/H165A double mutant. Negative: non-transfected cells. Scale bar: 100 µm.
