# Supplementary material for: SARS-CoV-2 nsp1 mediates broad inhibition of translation in mammals
Source: Cell Rep. Author manuscript; Available in PMC 2026 Jun 8. (PMC13245623; doi:10.1016/j.celrep.2025.115696)
Supplement: FigureS3 [file NIHMS2171571-supplement-FigureS3.pdf]

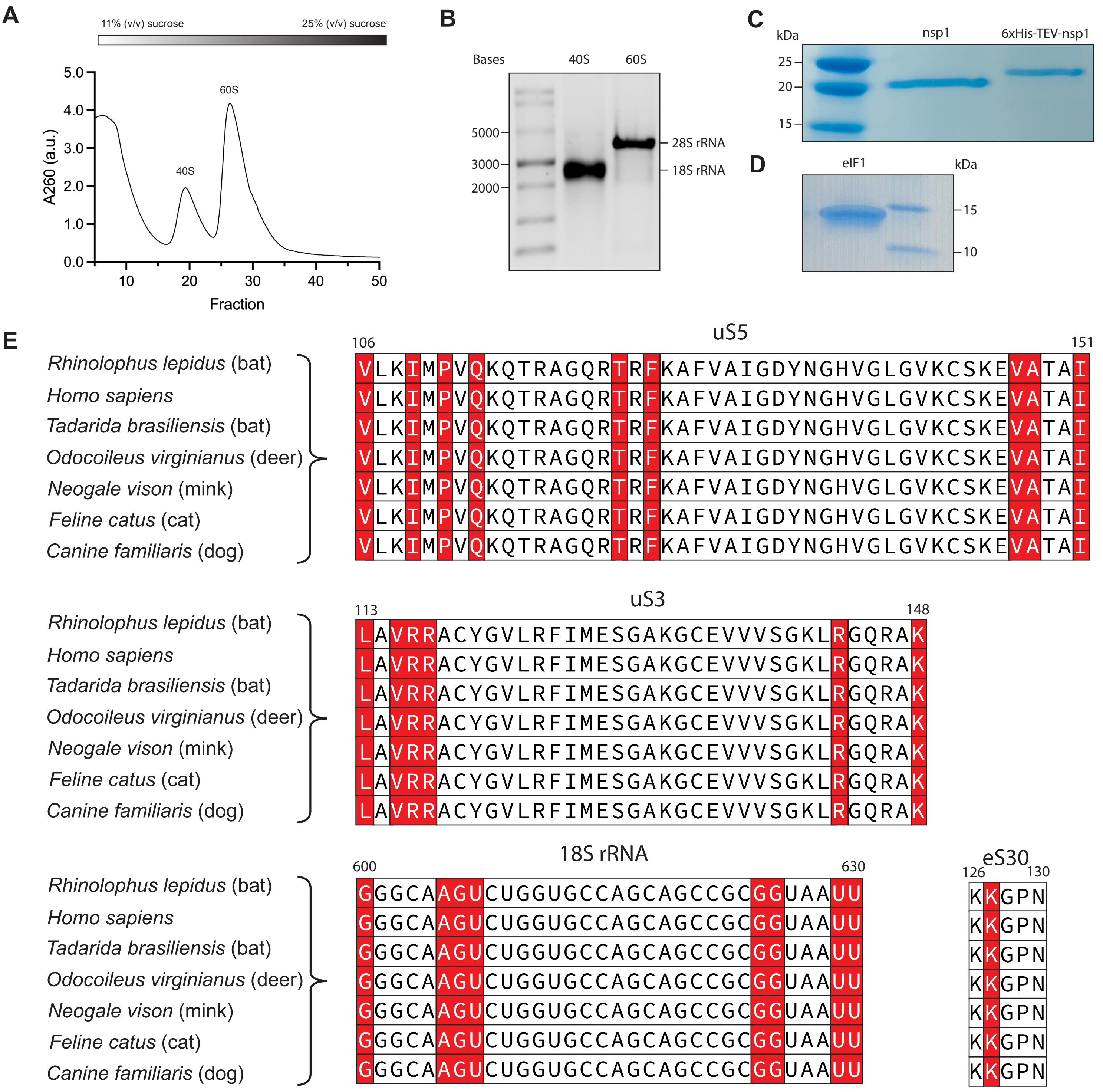

A
11% (v/v) sucrose
25% (v/v) sucrose
A260 (a.u.)
5.0
4.0
3.0
2.0
1.0
0.0
40S
60S
10
20
30
40
50
Fraction
B
Bases
40S
60S
5000
3000
2000
28S rRNA
18S rRNA
C
kDa
nsp1
6xHis-TEV-nsp1
25
20
15
D
eIF1
kDa
15
10
E
uS5
106
151
Rhinolophus lepidus (bat)
Homo sapiens
Tadarida brasiliensis (bat)
Odocoileus virginianus (deer)
Neogale vison (mink)
Feline catus (cat)
Canine familiaris (dog)
VLKIMPVQKQTRAGQRTRFKAFVAIGDYNGHVGLGVKCSKEVATAI
uS3
113
148
LAVRRACYGVLRFIMESGAKGCEVVVSGKLRGQRAK
18S rRNA
600
630
GGGCAAGUCUGGUGCCAGCAGCCGCGGUAAUU
eS30
126
130
KKGPN
